# Supplementary material for: Multi‐dataset identification of innovative feature genes and molecular mechanisms in keratoconus
Source: J Cell Mol Med. 2024 Sep 19;28(18):e70079. doi: 10.1111/jcmm.70079 (PMC11412914; doi:10.1111/jcmm.70079)
Supplement: Supplementary file 2 — Material S2. [file JCMM-28-e70079-s002.zip › 7.GSEA analysis/4.MYRF/KEGG_NOD_LIKE_RECEPTOR_SIGNALING_PATHWAY.html]

Details for gene set KEGG\_NOD\_LIKE\_RECEPTOR\_SIGNALING\_PATHWAY[GSEA]

|  || Dataset | MYRF.group.cls#h\_versus\_l |
| Phenotype | group.cls#h\_versus\_l |
| Upregulated in class | l |
| GeneSet | KEGG\_NOD\_LIKE\_RECEPTOR\_SIGNALING\_PATHWAY |
| Enrichment Score (ES) | -0.5341022 |
| Normalized Enrichment Score (NES) | -1.7424049 |
| Nominal p-value | 0.035714287 |
| FDR q-value | 0.30873334 |
| FWER p-Value | 0.289 |
Table: GSEA Results Summary

  

Fig 1: Enrichment plot: KEGG\_NOD\_LIKE\_RECEPTOR\_SIGNALING\_PATHWAY      
 Profile of the Running ES Score & Positions of GeneSet Members on the Rank Ordered List

  

| SYMBOL | TITLE | RANK IN GENE LIST | RANK METRIC SCORE | RUNNING ES | CORE ENRICHMENT || 1 | MEFV | na | 3441 | 0.275 | -0.0715 | No |
| 2 | CARD6 | na | 6303 | 0.183 | -0.1355 | No |
| 3 | MAPK9 | na | 8409 | 0.129 | -0.1831 | No |
| 4 | MAPK12 | na | 8572 | 0.125 | -0.1751 | No |
| 5 | CASP8 | na | 9334 | 0.109 | -0.1860 | No |
| 6 | NAIP | na | 9445 | 0.107 | -0.1784 | No |
| 7 | NLRP1 | na | 10212 | 0.092 | -0.1912 | No |
| 8 | MAPK11 | na | 10606 | 0.085 | -0.1940 | No |
| 9 | MAPK13 | na | 10951 | 0.079 | -0.1959 | No |
| 10 | PYCARD | na | 11234 | 0.075 | -0.1965 | No |
| 11 | CARD8 | na | 11471 | 0.071 | -0.1962 | No |
| 12 | XIAP | na | 11781 | 0.066 | -0.1983 | No |
| 13 | MAPK14 | na | 11965 | 0.064 | -0.1972 | No |
| 14 | HSP90AA1 | na | 12360 | 0.059 | -0.2026 | No |
| 15 | MAPK10 | na | 12440 | 0.057 | -0.1991 | No |
| 16 | PYDC1 | na | 12878 | 0.052 | -0.2065 | No |
| 17 | SUGT1 | na | 12880 | 0.052 | -0.2013 | No |
| 18 | IKBKB | na | 12994 | 0.050 | -0.1994 | No |
| 19 | CARD9 | na | 13392 | 0.045 | -0.2063 | No |
| 20 | CHUK | na | 14348 | 0.034 | -0.2304 | No |
| 21 | MAPK3 | na | 14382 | 0.034 | -0.2280 | No |
| 22 | CARD18 | na | 15788 | 0.020 | -0.2665 | No |
| 23 | CCL11 | na | 15898 | 0.019 | -0.2678 | No |
| 24 | PSTPIP1 | na | 17337 | 0.006 | -0.3087 | No |
| 25 | BIRC2 | na | 18537 | -0.006 | -0.3427 | No |
| 26 | MAPK8 | na | 18612 | -0.006 | -0.3442 | No |
| 27 | TAB1 | na | 19282 | -0.014 | -0.3621 | No |
| 28 | CASP5 | na | 19389 | -0.015 | -0.3636 | No |
| 29 | MAPK1 | na | 20406 | -0.027 | -0.3903 | No |
| 30 | CASP1 | na | 20415 | -0.027 | -0.3878 | No |
| 31 | HSP90AB1 | na | 20726 | -0.030 | -0.3937 | No |
| 32 | IKBKG | na | 21687 | -0.042 | -0.4171 | No |
| 33 | TRIP6 | na | 21714 | -0.042 | -0.4136 | No |
| 34 | IL18 | na | 22163 | -0.048 | -0.4217 | No |
| 35 | CCL8 | na | 22516 | -0.052 | -0.4266 | No |
| 36 | TAB2 | na | 22545 | -0.053 | -0.4220 | No |
| 37 | NOD1 | na | 23048 | -0.060 | -0.4304 | No |
| 38 | TAB3 | na | 23218 | -0.063 | -0.4289 | No |
| 39 | TRAF6 | na | 23526 | -0.068 | -0.4309 | No |
| 40 | BIRC3 | na | 24271 | -0.080 | -0.4443 | No |
| 41 | HSP90B1 | na | 24682 | -0.088 | -0.4472 | No |
| 42 | MAP3K7 | na | 24724 | -0.088 | -0.4395 | No |
| 43 | NFKBIB | na | 25462 | -0.102 | -0.4504 | No |
| 44 | NLRC4 | na | 26678 | -0.128 | -0.4725 | No |
| 45 | CCL13 | na | 27880 | -0.157 | -0.4913 | No |
| 46 | NFKB1 | na | 28241 | -0.168 | -0.4847 | No |
| 47 | CCL2 | na | 28806 | -0.185 | -0.4822 | No |
| 48 | CCL7 | na | 30606 | -0.246 | -0.5092 | Yes |
| 49 | CCL5 | na | 31086 | -0.268 | -0.4959 | Yes |
| 50 | RELA | na | 31135 | -0.271 | -0.4699 | Yes |
| 51 | NOD2 | na | 31442 | -0.288 | -0.4496 | Yes |
| 52 | NFKBIA | na | 31525 | -0.292 | -0.4224 | Yes |
| 53 | RIPK2 | na | 33268 | -0.416 | -0.4306 | Yes |
| 54 | TNFAIP3 | na | 33327 | -0.422 | -0.3896 | Yes |
| 55 | IL6 | na | 33353 | -0.425 | -0.3473 | Yes |
| 56 | CXCL2 | na | 34010 | -0.524 | -0.3132 | Yes |
| 57 | NLRP3 | na | 34210 | -0.574 | -0.2609 | Yes |
| 58 | IL1B | na | 34248 | -0.586 | -0.2027 | Yes |
| 59 | TNF | na | 34328 | -0.609 | -0.1434 | Yes |
| 60 | CXCL1 | na | 34529 | -0.693 | -0.0790 | Yes |
| 61 | CXCL8 | na | 34657 | -0.838 | 0.0020 | Yes |
Table: GSEA details [plain text format]

  

Fig 2: KEGG\_NOD\_LIKE\_RECEPTOR\_SIGNALING\_PATHWAY      
 Blue-Pink O' Gram in the Space of the Analyzed GeneSet

  

Fig 3: KEGG\_NOD\_LIKE\_RECEPTOR\_SIGNALING\_PATHWAY: Random ES distribution      
 Gene set null distribution of ES for **KEGG\_NOD\_LIKE\_RECEPTOR\_SIGNALING\_PATHWAY**
